# Efficacy of 14-day concomitant quadruple therapy and 14-day high-dose dual therapy on *H. pylori* eradication 

**Published:** 2022

**Authors:** Behsood Yadollahi, Seyed Mohammad Valizadeh Toosi, Zohreh Bari, Hafez Fakheri, Iradj Maleki, Tarang Taghvaei, Vahid Hosseini, Arash Kazemi, Hajar Shokri-Afra

**Affiliations:** *Non-communicable Diseases Institute, Gut and Liver Research Center, Mazandaran University of Medical Sciences, Sari, Iran *

**Keywords:** H. pylori, High-Dose Dual Therapy, Concomitant Quadruple Therapy, Eradication

## Abstract

**Aim:**

We compared the efficacy of two different regimens for H*. pylori* eradication in areas with high antibiotic resistance.

**Background:**

Helicobacter *pylori* (*H. pylori*) is a gram-negative bacillus that has a strong association with chronic gastritis and peptic ulcer disease. Different regimens with varying degrees of effectiveness have been used for *H. pylori* eradication.

**Methods:**

The current randomized controlled trial (RCT) randomly assigned 217 patients who had indications for H*. pylori* eradication therapy to two groups. One group were administered concomitant quadruple therapy (pantoprazole 40 mg, amoxicillin 1 gr, clarithromycin 500 mg, and metronidazole 500 mg every 12 hours) for 14 days, and the second group received 14 days of high-dose dual therapy, consisting of esomeprazole 40 mg BID and amoxicillin 1g TDS. *H. pylori* eradication was assessed eight weeks after the end of treatment.

**Results:**

*H. pylori* eradication rates by PP analysis for 14 days concomitant quadruple therapy and high-dose dual therapy were 88.6% (95% CI, 80.3−92.8) and 82.2% (95% CI, 74.8–89.5), respectively (*p* = 0.19). According to intention-to-treat (ITT) analysis, the eradication rates were 81.6% (95% CI, 74.5−88.6) and 80.6% (95% CI, 73–88.1), respectively (*p* = 0.58). Overall drug side effects were 20.8% in high-dose dual therapy and 49.6% in concomitant quadruple therapy (*p* < 0.001).

**Conclusion:**

Fourteen days concomitant quadruple therapy can be considered as a relatively acceptable regimen for *H. pylori* eradication in areas with high clarithromycin and metronidazole resistance. It seems that high-dose dual therapy could be a promising alternative regimen in these areas.

## Introduction

 Nearly half of the world’s population are infected with *H. pylori*. The relevant organism plays an important role in the development of upper gastrointestinal tract diseases, such as peptic ulcer disease, gastric adenocarcinoma, and MALT lymphoma (1). *H. pylori* eradication accelerates the healing of peptic ulcers and prevents their recurrence (2). Despite many efforts to achieve a proper *H. pylori* eradication regimen in recent decades, successful ones are few (3-5). Up to 20% of treatment failure in the eradication of *H. pylori* has been reported in some meta-analyses (6-8). Thus, the need for new clinical trials to determine an appropriate treatment is obvious.

The most important challenge in treating *H. pylori* infection is antibiotic resistance. In areas where antibiotic resistance to clarithromycin is high (more than 15%), it is recommended that a bismuth- or non-bismuth-based concomitant quadruple regimen be used for 14 days (9). Over the past 20 years, the resistance of this bacterium to antibiotics has been increasing in different parts of Iran (10). In their 2015 systematic review, Khademi et al. showed that in Iran, average *H. pylori* antibiotic resistance rates to metronidazole, clarithromycin, amoxicillin, and furazolidone were 66.6%, 22.4%, 16.0%, and 21.6%, respectively (11).

Concomitant non-bismuth-based quadruple therapy is a treatment with simultaneous administration of a proton pump inhibitor (PPI), amoxicillin, metronidazole, and clarithromycin. Previous studies from different countries as well as Iran have reported acceptable *H. pylori* eradication with 14-day concomitant quadruple therapy (12-18). In Spain in 2013, the per-protocol eradication rate of the concomitant quadruple regimen was 96.1% (13). In a multicenter study in Italy in 2013, the per-protocol eradication rate of five-day concomitant quadruple regimen was 91.6% (12). In another study in Italy in 2014, the per-protocol eradication rate for 14-day concomitant quadruple therapy was 95%, while a five-day concomitant quadruple regimen did not produce the desired result (15). Per-protocol eradication rates in concomitant 10- and 14-day quadruple regimens in Korea were reported to be 95.6% and 98.5%, respectively (14). Another study in Korea in 2018 reported the per-protocol eradication rate of a 10-day concomitant quadruple regimen was 95.5% (16). Studies from Taiwan, a country with low drug resistance of *H. pylori* infection, have also reported acceptable eradication rates for 7-, 10-, and 14-day concomitant quadruple therapy (16, 19).

Two studies from Iran have also evaluated the effectiveness of concomitant quadruple therapy. In 2015, Alhooei et al. reported an unacceptable eradication rate (83.1%) with 10 days of concomitant therapy (17). In 2020, Bari et al. reported *H. pylori* eradication rates of 88.8% and 92.6% for 10- and 12-day concomitant quadruple therapies, respectively, based on ITT analysis (17, 18). 

*H. pylori* infection is antibiotic resistance. According to the Maastricht V consensus, in areas of high dual clarithromycin and metronidazole resistance (greater than 15%), bismuth quadruple therapy is the recommended first-line treatment for *H. pylori *infection (9).

As the results of Iranian studies on non-bismuth-based quadruple therapy in the eradication of *H*.* pylori* infection are close to 90%, it is thought that dual drug resistance to clarithromycin and metronidazole is the weak point in achieving excellent eradication rates by non-bismuth-based quadruple therapy in this region.

Because of the high prevalence of clarithromycin and/or metronidazole-resistant strains of *H. pylori *in the studied region (11), the high-dose dual therapy of amoxicillin plus a proton pump inhibitor may be an alternative regimen for overcoming this problem (20). Various studies comparing high-dose dual therapy with other *H. pylori* eradication regimens have shown that high-dose dual therapies have good efficacy with better drug compliance, lower costs, and fewer side effects (21-25). However, these studies have been conducted mainly in areas with low antibiotic resistance. As is known, Iran is a country with high rates of drug-resistant *H*. *pylori* infection (11, 26). According to the Maastricht V guidelines, the standard regimen in such regions is bismuth- or non-bismuth-based concomitant quadruple therapy. Moreover, a high-dose dual therapy of amoxicillin + PPI is recommended for use in these areas in cases of bismuth inaccessibility or intolerance. Therefore, the current study purposed to compare the *H. pylori* eradication rates of a 14-day concomitant quadruple therapy with those of a 14-day high-dose dual therapy of PPI plus amoxicillin. 

## Methods

In this clinical trial, adult patients with symptoms of long-standing dyspepsia accompanied by warning signs and symptoms (including age > 45 years or having weight loss, iron deficiency anemia, hematemesis or melena and dyspepsia refractory to proton pump inhibitor therapy, and/or a family history of gastric cancer in first degree relatives) underwent upper endoscopy. Then, patients who were *H. pylori* positive and had endoscopic findings of gastric ulcer or erosion, duodenal ulcer, or duodenitis and/or histologic evidence of intestinal metaplasia were enrolled in the study. The exclusion criteria were pregnant or nursing women; those with a history of upper GI surgery; major cardiac, lung, liver or renal diseases; a history of previous *H. pylori* eradication regimen; or a history of known side effects to the protocol drugs. 

Endoscopy was performed by one of the three collaborating gastroenterologists using a Pentax endoscope (version: EG2985). We evaluated *H. pylori* infection by two biopsies taken from the antrum and body using the Rapid Urease Test (RUT) method (product of Shim-enzyme Company) and/or Giemsa staining of the biopsy specimens. In patients with abnormal endoscopic findings, pathological specimens were obtained from the visible abnormal lesions. Finally, according to the inclusion and exclusion criteria, 217 patients were included in the study. Informed consent was obtained from all participants. Patients were allocated to two different regimens for *H. pylori* eradication. The first group (PACM group) received 14 days of a concomitant quadruple regimen composed of: (pantoprazole 40 mg, amoxicillin 1 g, clarithromycin 500 mg, and metronidazole 500, all twice daily), and the second group (EA group) received high-dose dual therapy composed of esomeprazole (Ezonium of Dr. Abidi Pharmaceutical Company) 40 mg BID and amoxicillin 1 g TDS for 14 days.

**Table 1:** Demographic characteristics and endoscopic findings of patients of two groups

Study variables	Concomitant quadruple therapy	High dose dual therapy	P value
Sex Male/female	55/59	49/54	0.92
Age (year)	45.5 ± 14.4	51.6 ± 12.2	0.001
ASA usage	20 (17.5%)	13 (12.6%)	0.31
History of smoking	15 (13.2%)	16 (15.5%)	0.61
Endoscopic findings			
Peptic ulcer disease (DU or GU)	66/114 (57.8%)	55/103(53.4%)	
Erosive gastritis and duodenitis	47/114(38.9%)	47/103 (45.6%)	
Intestinal metaplasia	1 (0.9%)	1 (1%)	

**Table 2:** Frequency and severity of side effects and rate of treatment acceptance in both groups

Treatment regimens	Concomitant quadruple	High dose dual	P value
Without complication	58 (50.8%)	81 (78.6%)	0.001
Side effects			
Mild	49 (42.9%)	22 (21.3%)	0.001
Moderate	5 (4.4%)	0 (0%)	0.001
Severe	2 (1.7%)	0 (0%)	0.001
Compliance	105 (92.1%)	101 (98%)	0.046

Each patient completed a questionnaire including demographic information of age, sex, history of aspirin usage and cigarette smoking as well as endoscopic findings, pathology results, and data on *H. pylori* infection.

Eight weeks after treatment, *H. pylori* eradication was evaluated by *H. pylori* stool antigen test and/or RUT and pathology in patients who needed re-endoscopy after treatment.

During treatment, drug side effects were recorded for all patients. Compliance of patients for drug consumption was assessed during the study. The compliance was considered good if the patients consumed more than 80% of the prescribed drugs. Those who took 60% to 80% of the drugs had intermediate compliance, and those who consumed less than 60% were categorized as low compliance.

The data were analyzed by SPSS-24 software. Central and dispersion indices were used to describe the data, and chi-square tests were used for qualitative and t-tests for quantitative data. A *p*-value < 0.05 was considered statistically significant.

To calculate eradication rate based on intention to treat, all patients who were initially included in the study were analyzed. To calculate eradication rate based on per protocol, only patients who followed all the steps of the study protocol and took more than 80% of the drugs were included in the statistical analysis. 

The current study proposal was approved by the scientific members of the Gut and Liver Research Center and the Ethics Committee of Mazandaran University of Medical Sciences (ethics code: IR.MAZUMS.IMAMHOSPITAL.REC.1399.7090). Moreover, this study was registered in the Iranian Registry of Clinical Trials with the IRCT number IRCT20131124015510N4. 

## Results

A total of 217 patients were included in the study. One hundred and fourteen patients received 14-day concomitant quadruple therapy, and 103 patients received the 14-day high-dose dual therapy regimen. The percentages of female participants in the concomitant and dual therapy groups were not statistically different (51.7% and 52.4%, respectively; *p* = 0.92) 

Patient mean age in the concomitant and dual therapy groups was 45.5 ± 14.4 years and 51.6 ± 12.3 years, respectively. There was no significant difference between the two groups in terms of ASA consumption or history of smoking. The most common endoscopic finding in all patients was peptic ulcer disease (Table 1). 

**Flow chart 1:**
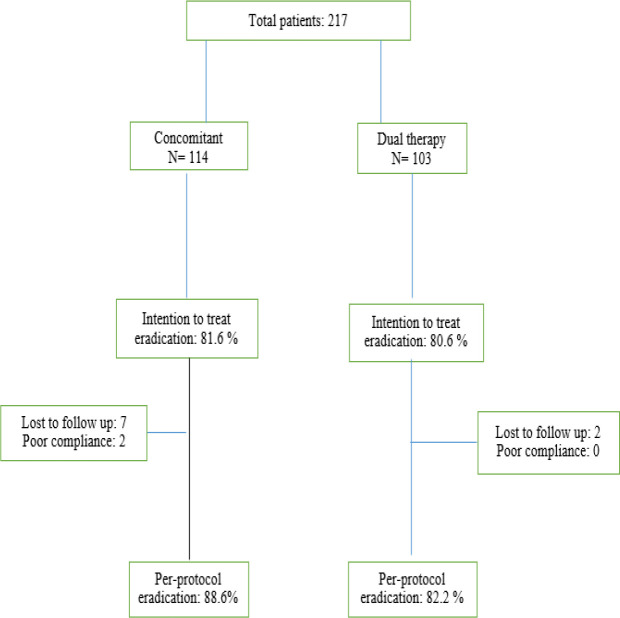
Eradication rates achieved by both groups according to intention to treat and per-protocol analyses

Based on ITT analysis, the *H*. *pylori* eradication rate in the concomitant quadruple therapy regimen was 81.6% (95% CI, 74.5% -88.6%) and 80/6% in the high dose dual therapy (95% CI, 73% -88.1%) (*p* = 0.58). Two hundred and six of the 217 patients completed the study. Nine patients in the concomitant regimen and 2 patients in the dual therapy group did not complete the study protocol due to improvement in clinical symptoms and lack of cooperation in conducting the final test to evaluate *H.* *pylori* eradication. Therefore, according to per-protocol analysis, the rate of *H.* *pylori* eradication in the concomitant quadruple and high dose dual therapy was 88.6% (95% CI, 80.3% -92.8%) and 82.2% (95% CI, 74.8% -89.5%), respectively (*p* = 0.19) (Flow Chart 1). In none of the treatment groups was any correlation between endoscopic findings and treatment success rate observed.

The rates of drug side effects were 49.6% and 20.8% in the concomitant quadruple and high-dose dual therapy regimens, respectively (*p* < 0.001). However, most side effects were mild in the concomitant regimen, with only 1.8% of patients reporting severe side effects, whereas the high-dose dual therapy regimen had no severe side effects. Moreover, the rate of treatment acceptance was 92.1% in the concomitant group and 98.1% in the high dose dual therapy group (*p* = 0.046) (Table 2).

## Discussion

According to the current results, the *H*. *pylori* eradication rates based on per-protocol analysis in the concomitant quadruple therapy regimen and the high dose dual therapy regimen were 88.6% and 82.2%, respectively. Based on the results of a study by Graham et al., in evaluating the response rate of *H.* *pylori* eradication regimen (based on PP analysis), the success rate of ≥ 95% is considered as an excellent eradication rate, 90-95% is considered as good, 85-89% as relatively good (borderline acceptable), and <85% is considered as unacceptable borderline.

Therefore, in the current study, the therapeutic response rate of the 14-day concomitant quadruple therapy regimen was in the acceptable range and considered relatively good; however, the high-dose dual therapy regimen response rate was in the range of unacceptable borderline.

This study is the first to evaluate the *H. pylori* eradication rate of the high-dose dual therapy regimen in Iran. Because of the increasing prevalence of clarithromycin and/or metronidazole-resistant strains of *H.* *pylori*, the high-dose dual therapy of amoxicillin plus PPI regimen was suggested as an alternative to overcome this problem (20).

Various studies in other parts of the world have examined the effectiveness of the high-dose dual therapy. Zullo et al. conducted a study in Italy and reported an *H.* *pylori* eradication rate of 87.5% achieved by the high-dose dual therapy regimen of omeprazole and amoxicillin (20). 

In a study in Taiwan in 2015, high-dose dual therapy regimen of rabeprazole plus amoxicillin achieved a 95.3% PP eradication rate as the first line treatment for *H*. *pylori* infection (24). Contrary to these successful results for the eradication of *H. pylori, *studies from Korea and China, similar to the results of the current study, reported the ineffectiveness of a high-dose dual therapy regimen as a first-line treatment for eradication of *H. pylori* infection; however, the high-dose dual therapy in their studies as well as the current one showed excellent acceptance and minimal side effects (23, 27, 28) (Table 2).

Conversely, Yu et al. reported a high level of eradication for high-dose omeprazole + amoxicillin dual therapy in China in 2019 (> 90%). It is important to mention that antibiotic resistance to amoxicillin in this study was 0% (25). In another clinical trial in China in the same year, Yang et al. reported an eradication rate of 91.1% achieved by a modified dual therapy regimen (omeprazole + amoxicillin) for *H. pylori* infection. *H.* *pylori* antibiotic resistance to amoxicillin in the current study, similar to that of Yu et al., was zero, while resistance to clarithromycin was 29.7% and to metronidazole was 96.6% (22%).

Contradictory results regarding the effectiveness of high-dose dual therapy in some previous studies may be largely attributed to differences in the dosage and frequency of PPI-amoxicillin administration. High dosage PPI (27, 29) and increasing the duration of treatment (22, 30) significantly improve the success rate of *H. pylori* eradication (31). Administration of PPI-amoxicillin less than four times a day did not meet treatment expectations and was unacceptable for eradicating *H. pylori* infection (32-34). Sugimoto et al. examined the relationship between the frequency of PPI administration and its inhibitory effects on intragastric acid secretion. By measuring the pH values in the stomach over a 24-hour period, they found that administering different amounts of PPI led to different pH values in the stomach being obtained. They administered 40 mg of esomeprazole once (pH = 4.8), twice (pH = 5.7) and four times daily (pH = 6) (35). Thus, the administration of PPI and amoxicillin 2 and 3 times daily in the dual therapy regimen may not be ideal for maximizing the pharmacokinetic and pharmacodynamic effects of drugs in the eradication of *H. pylori* infection (22). Based on the above data, the high *H. pylori* drug resistance to amoxicillin in Iran (11, 26) as well as the low frequency of administration of esomeprazole in this study (40 mg twice daily) may explain the failure of our high-dose dual therapy regimen for *H. pylori* eradication.

In all *H. pylori* eradication regimens, beyond the main goal of treatment which is to achieve an eradication rate above 90%, the next step is to reduce the side effects of therapy, improve tolerance and ensure proper use of the drugs by the patients (10). The results of the current study showed that the high-dose dual therapy regimen had a much lower rate of side effects (21.3%) than the concomitant quadruple therapy regimen (49%) (*p* < 0.001). In addition, all drug side effects were mild in the dual therapy regimen. The rate of drug acceptance in the concomitant quadruple therapy was 92.1%, while this rate was significantly better (98%) in patients treated with the high-dose dual therapy regimen (*p* = 0.04).

Lower treatment costs combined with fewer pills required in the high-dose dual therapy regimen were other advantages of this regimen over the 14-day concomitant quadruple regimen.

The main limitation of the present study was the unavailability of *H. pylori* cultures and antibiotic susceptibility/resistance tests. However, the strength of this study is that for the first time, the effectiveness of the high-dose dual therapy regimen as the first-line treatment regimen for *H. pylori* eradication in Iran, a region with high resistance to metronidazole and clarithromycin, was evaluated.

In conclusion, fourteen days concomitant quadruple therapy could be considered as a relatively acceptable regimen in areas with high clarithromycin and metronidazole resistance. However, the high-dose dual therapy regimen of esomeprazole + amoxicillin has borderline unacceptable response rate as a first-line therapy in this area. It seems that increasing the dose of PPI can increase the *H. pylori* eradication rate. Further studies are needed.

## Conflict of interests

The authors declare that they have no conflict of interest.
